# Molecular timekeepers: the curious alliance of redox, repair, and protective proteins in preserving seed longevity

**DOI:** 10.1007/s44297-026-00064-9

**Published:** 2026-01-30

**Authors:** Arup Das, Manoj Majee

**Affiliations:** BRIC-National Institute of Plant Genome Research, Aruna Asaf Ali Marg, New Delhi, 110067 India

**Keywords:** Seed longevity, Orthodox seeds, Heat-shock protein, LEA protein, DNA repair, MSR, PIMT

## Abstract

Seed longevity—the ability of seeds to remain viable over time—is an evolutionary masterpiece, ensuring plant survival across generations and in the face of environmental variability. Desiccation-tolerant (orthodox) seeds, representative of most crop species, possess the ability to survive programmed drying during maturation, thereby entering a metabolically inactive state. This anhydrobiotic state serves to prolong embryo viability and shield against adverse environmental conditions. While programmed drying is essential for seed preservation, it can result in oxidative and macromolecular damage, which is exacerbated by fluctuations in temperature and humidity during storage. Consequently, this cumulative damage to deoxyribonucleic acid, proteins, and cellular structures can jeopardize seed viability if not adequately repaired. Seed longevity is therefore dependent not merely on passive resistance by molecular stabilizers but on an active repair mechanism that is initiated upon rehydration. The interplay between redox homeostasis, damage repair, and cellular protective proteins forms the cornerstone of seed longevity, helping seeds retain their ability to germinate. This review delves into the converging roles of redox homeostasis, repair, and protective proteins in governing the longevity of seeds. By unraveling how these components cooperate and communicate, we gain deeper insights into the natural strategies that seeds employ to delay aging. Exploring the molecular underpinnings of seed longevity offers substantial novel genetic targets for developing crops with improved resistance to evolving climates and provides crucial insights for the conservation of plant germplasm.

## Introduction

Seeds serve as the principal reproductive structure for most plant species, underpinning the foundation of both agriculture and ecological environments. The structural organization of a seed represents a sophisticated example of natural design, intended to safeguard and sustain the plant embryo it contains. However, seeds, much like all living entities, also undergo aging, gradually losing vigor and viability until they eventually die. Seed longevity is defined as the time frame from maturation to the point at which seed viability is lost during dry storage. Longevity is closely related to its germination success, making the improvement of this characteristic crucial for effective long-term seed preservation and agricultural advancement [1]. It is also crucial in ex situ conservation strategies for plant genetic resources, serving as an “insurance policy” against biodiversity loss [2]. The inherent capacity of seeds to remain viable is one of the adaptive traits that facilitates plant populations to disperse in time and space [3].

In contrast to recalcitrant seeds, which lack significant mechanisms to prevent rapid deterioration and are released from the parent plant with high moisture content and metabolic activity, orthodox seeds develop desiccation tolerance during the latter stages of maturation. This acquired tolerance grants them the capacity to maintain viability for extended durations in a quiescent, dry state. DT (orthodox) seeds are more prevalent in temperate regions and among major crop species, in contrast to the greater abundance of recalcitrant seeds in tropical latitudes [4]. The longevity of orthodox seeds displays considerable interspecific variability, with seed viability being maintained for periods ranging from several years to a millennium [5]. For instance, date palm seeds that were 2,000 years old, discovered at Herod’s Palace near Jerusalem, were successfully germinated, resulting in the development of viable trees [6]. Viable seeds of the sacred lotus, originating from northeastern China, have demonstrated a longevity of approximately 1,300 years [7].

Over recent decades, substantial research has focused on elucidating the complexities of seed longevity, aiming to reveal the factors contributing to the extended viability of certain seeds compared to others. Beyond cellular modifications, including chromatin condensation, cell wall folding, and thylakoid dismantling within chloroplasts [8], three principal mechanisms collaborate to counteract the detrimental impacts of desiccation and determine seed longevity. These are (1) the prevention of oxidative damage through the accumulation of antioxidant compounds, coupled with the coordinated suppression of fundamental metabolic processes during dehydration [9]; (2) the stabilization of cellular membranes and proteins by employing non-reducing sugars, LEA proteins, and HSPs, which simulate a hydrated state in dry molecules by forming hydrogen bonds with polar residues on proteins and membrane phospholipids—a phenomenon termed the “water replacement hypothesis” [10]; and (3) the implementation of effective repair systems for DNA and protein damage upon the rehydration of the seed [11]. The integrated regulation of these strategies is essential for preserving seed viability and fostering extended longevity. In this review, we aim to synthesize recent advances on redox-regulated mechanisms influencing seed longevity and propose a conceptual framework linking redox homeostasis, macromolecular repair pathways, and protective proteins such as LEA proteins and HSPs. By defining these interconnections, we intend to provide a unified perspective that may guide future research toward understanding and enhancing seed longevity. This discussion primarily examines the synergistic mechanisms governing seed longevity, with a particular emphasis on the functional interplay between redox homeostasis, macromolecule repair systems, and the DT protective proteins.

### Drying without dying: more than just water loss

**Fig. 1:**
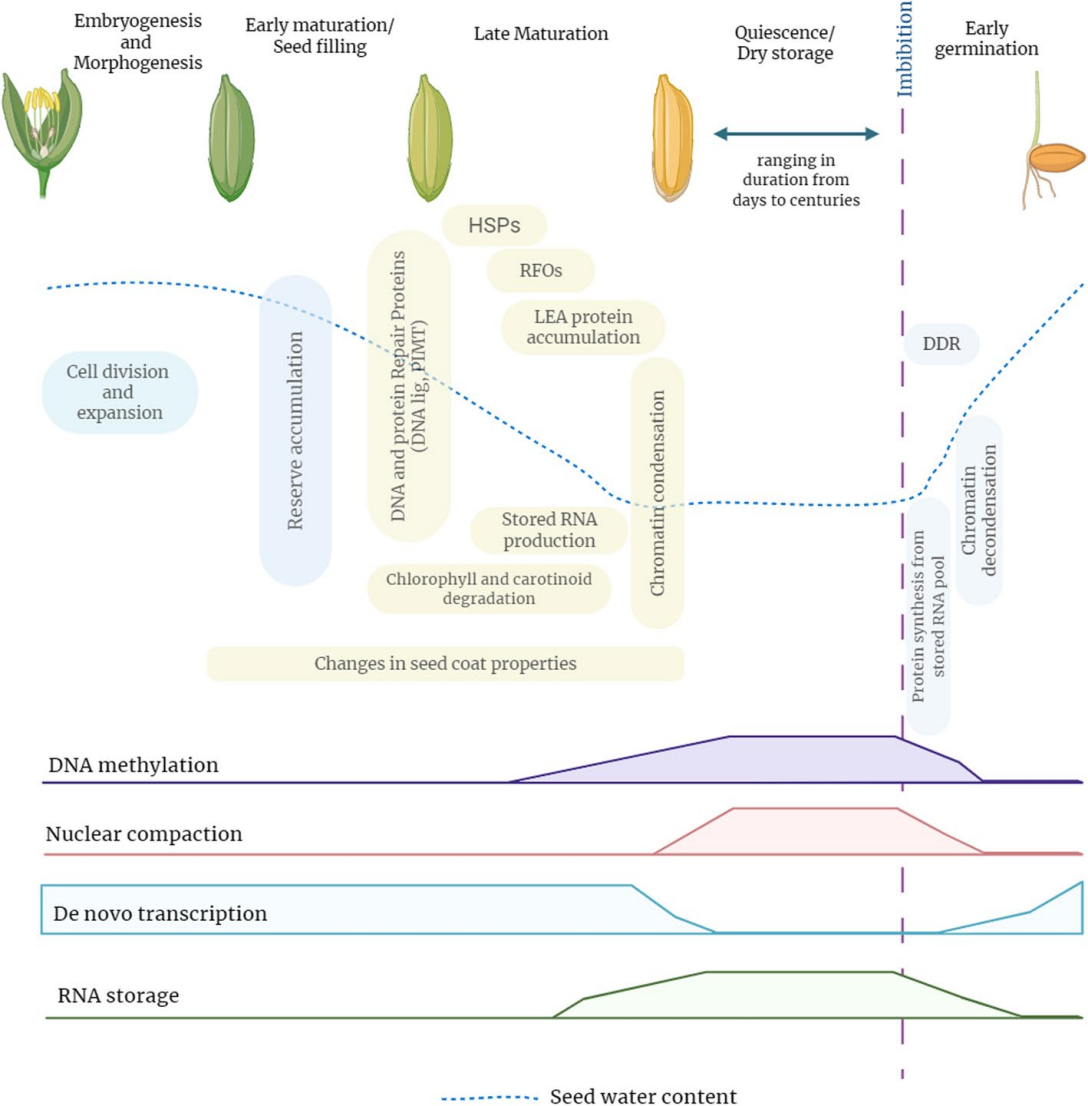
A simplified timeline illustrating late maturation changes in orthodox seeds. Key events include the accumulation of storage compounds, protective proteins (e.g., LEA, HSPs), DNA and protein repair proteins, significant nuclear changes (chromatin condensation), and a dramatic reduction of seed water content. Elevated DNA compaction, induced by DNA methylation, diminishes the susceptibility of DNA to damage and restricts de novo transcription during the quiescent phase. Abbreviations: DNA, deoxyribonucleic acid; HSP, heat-shock protein; LEA, late embryogenesis abundant; RFO, non-reducing oligosaccharide; DDR, DNA damage repair; RNA, ribonucleic acid

The capacity of survival through dehydration, initially observed as a crucial adaptation in early photoautotrophic organisms, facilitated their persistence in terrestrial environments characterized by significant fluctuations in water availability [12]. With the evolutionary advancement of land plants, particularly vascular species, this trait became predominantly confined to the protection of seeds and pollens, while the mature vegetative forms remain susceptible to desiccation [13]. Consequently, seeds have evolved complex, layered mechanisms to endure and maintain viability in a dehydrated condition. Upon concluding a sequence of cell division and differentiation, seed development advances to the maturation phase, characterized by early (seed filling) and late (maturation drying) maturation periods [14]. During the maturation phase, orthodox seeds undergo a significant dehydration process, losing up to 95% of their water content and entering a state of dormancy (Fig. 1). This dehydration causes the cytoplasm to transition from a fluid to a glassy state, where the molecular mobility and relaxation rates of molecules are significantly impeded, thereby inducing metabolic quiescence and enhancing longevity [9]. This characteristic enables seeds to preserve their viability amidst substantial environmental variability [15].

The underlying mechanisms of desiccation tolerance are initiated during the late maturation stage of seed development and involve the accumulation of various protective molecules, including LEA proteins, sHSPs, RFOs, and diverse antioxidants (Fig. 1). The precise commencement of late seed maturation remains a subject of debate; however, a practical indicator is the cessation of embryo and/or endosperm expansion and the conclusion of storage reserve accumulation [3]. While this phase typically starts after seed filling is complete, in certain species—such as *Arabidopsis*—these events may begin earlier, suggesting a degree of concurrency between late maturation and the seed filling period [16, 17]. A prominent physiological change observed during late seed maturation is the catabolism of photosynthetic pigments (Fig. 1), including chlorophyll and carotenoids [18–20]. The transcription factor ABI3 plays a role in chlorophyll degradation by upregulating genes responsible for the initial stages of chlorophyll breakdown, including *NYC1 (NON-YELLOW COLORING1)* and *NOL (NON-YELLOW COLORING1-like)*, as well as *SGR2 (STAY-GREEN2)* [20, 21]. Nevertheless, the persistence of chlorophyll may adversely affect seed longevity as it leads to photo-oxidative stress. An alternate perspective suggests that chlorophyll degradation is critical for the release of phytyl tails; these serve as precursors for the synthesis of tocopherol, an antioxidant recognized for its role in promoting seed longevity [22]. While carotenoid metabolism is well-characterized in fruits and leaves [23], its role during seed maturation has received limited research attention. Furthermore, orthodox seeds exhibit RFO synthesis and accumulation as a hallmark of their late maturation stage. While it has been hypothesized that RFOs contribute to seed longevity by inhibiting crystallization or promoting glass formation during desiccation, current research does not substantiate these functions. Instead, RFOs might enhance seed longevity through their role in oxidative protection [24] or by modulating biotic stress signaling pathways, given their association with ABA-regulated defense mechanisms [25, 26].

### The double-edged sword

The maturation drying of seeds corresponds mechanistically to the drought stress [27]. While desiccation is critical for seed preservation, it simultaneously triggers macromolecular damage, thereby making successful germination contingent on a rapid cellular repair system upon rehydration. During the process of seed maturation drying, the elimination of hydration shells compromises the hydrogen bonding network, leaving hydrophilic residues susceptible to damage. This consequently induces partial unfolding in proteins and abnormal exposure of hydrophobic regions, thereby promoting their aggregation. Similarly, the reduction of bound water in membranes drives lipid bilayers from a liquid-crystalline phase to a rigid gel phase, leading to destabilization and leakage upon rehydration [28–30]. Furthermore, during desiccation, molecular oxygen interacts with intracellular transition metals (e.g., Fe^2+^, Cu^+^) via Fenton/Haber–Weiss reactions, leading to the generation of hydroxyl radicals (•OH) that oxidize macromolecules and consequently exacerbate cellular dysfunction [31]. Consequently, drying stress can also initiate a Maillard reaction, leading to the cross-linking and aggregation of proteins with sugars, thereby impeding the mobilization of storage proteins during germination [32]. Excessively rapid desiccation can lead to cellular damage within the embryo, manifesting as lipid peroxidation, thereby compromising cellular integrity and the germination capacity [33].

While desiccation effectively curtails the incidence of DNA damage, it simultaneously impedes the operation of repair mechanisms. As a result, genome damage can accumulate over prolonged dry dormancy. Additionally, desiccation/rehydration cycles can lead to genetic mutations, inhibited transcription and replication, and delayed growth and development [34]. In *Pyrus communis*, severe dehydration (approx. 2%−3% moisture content) was observed to elevate overall DNA methylation, which may impact its viability. Even moderate drying induced changes in methylation patterns, and prolonged storage resulted in further alterations to DNA methylation levels [35]. Thus essential for seed survival, desiccation paradoxically impairs DNA repair and epigenetic reprogramming. Cumulative DNA damage, mutations, and methylation alterations during prolonged dry storage emphasize the need for protective mechanisms that ensure genomic stability, seed vigor, and longevity.

### Seed aging

Seed aging, or seed deterioration, is commonly described as an irreversible, cumulative, and inexorable process [36] that can lead to the accumulation of cellular damage, resulting in delayed seedling emergence, reduced ability to withstand stress, and ultimately, loss of viability [37]. Seed viability is significantly shaped by a complex interplay between genetic factors and environmental influences experienced during seed development, maturation, and storage, ultimately impacting their physiological state. Environmental elements such as storage temperature, hydration levels, and light influence the stability and functional efficacy of repair and protective proteins and metabolites vital for maintaining seed viability. At sufficiently low temperatures (below 20 °C) and moisture levels (≤ 0.1 g H_2_O g^−1^ dry weight), the seed cytoplasm solidifies into a non-crystalline matrix that impedes molecular diffusion, consequently extending seed longevity by slowing the biochemical reactions responsible for deterioration [9, 38]. Humidity modulates the water content of seeds, which directly influences the mobility of macromolecules and the activity of metabolic and repair enzymes. High relative humidity raises seed moisture levels, activating metabolic processes (including respiration) that can exacerbate ROS generation [39, 40]. Even under dry conditions, seeds contain lipids that, due to their susceptibility to oxidation even at low humidity, serve as the predominant source of free radicals [41]. One of the most prominent physiological changes during seed aging is the accumulation of free radicals, a phenomenon exacerbated by adverse conditions, resulting in the accumulation of oxidation products of proteins, DNA, and lipids as the cellular environment becomes increasingly oxidized. Such redox imbalance particularly impairs DNA maintenance by inactivating or misfolding DNA repair proteins and by causing DNA strand breaks and base modifications [40]. Consequently, the aging stems from a deficit in the equilibrium between ROS generation and the efficacy of endogenous antioxidant defense mechanisms [42]. Non-enzymatic protein modifications, such as the Amadori and Maillard reactions, further contribute to the decline in viability during storage. The Amadori and Maillard reactions, initiated by reducing sugars, are notable contributors to seed aging. While seeds typically contain minimal reducing sugars, their levels increase post-maturation, facilitating reactions with cellular macromolecules. For instance, in mung bean seeds these processes have been correlated with lipid peroxidation and glucose hydrolysis. Specifically, the concentration of Amadori products rises during the early stages of seed aging, whereas Maillard products accumulate throughout the storage period [43]. The buildup of these products during storage has been shown to diminish seed vigor and longevity.

### The redox balancing act

Robust antioxidant machinery is identified as a key mechanism underlying desiccation tolerance [44]. Accordingly, DT seeds deploy highly sophisticated redox-protection networks that buffer ROS and maintain homeostasis across desiccation, dormancy, and germination, thereby keeping ROS at a level required for signaling without causing irreparable damage. The association between seed aging and the redox state is substantiated by findings that *Arabidopsis* ecotypes exhibiting elevated glutathione levels demonstrate enhanced seed longevity [45]. Glutathione governs the cellular redox state and exists in GSH and GSSG. However, GSH, the low-molecular-weight thiol-based antioxidant, is the most abundant water-soluble antioxidant in DT orthodox seeds [46]. It can neutralize ROS through direct scavenging or by providing electrons to ROS-detoxifying enzymes, including glutathione-S-transferases. With advancing seed age, the ratio of GSSG to GSH rises, signifying a progression toward a more oxidizing cellular environment and a decline in viability. The redox potential of the glutathione half-cell (E_GSSG/2GSH_) is recognized as a metric for assessing seed viability [47–49]. Tocopherols, among other antioxidants, are crucial for preserving seed viability by inhibiting non-enzymatic lipid oxidation throughout seed storage, and genotypes with impaired tocopherol biosynthesis exhibit diminished seed longevity [50, 51].

Emerging evidence from genome-wide and reverse genetics studies in *Arabidopsis thaliana* emphasizes the pivotal function of oxidative stress in seed aging, identifying genes involved in ROS metabolism and detoxification as related to seed longevity, including *DHAR1* and PSAD1 [52]. DHARs, a class of glutathione-dependent enzymes, are responsible for the regeneration of ascorbate, which is oxidized during detoxification of H_2_O_2_ by ascorbate peroxidase [53]. Renard et al. (2020) also reported that plants lacking DHAR1, but not DHAR2 or DHAR3, exhibit less seed longevity, likely due to reduced ROS detoxification [52]. This is consistent with DHAR gene expression profiles, with *DHAR1* being the most expressed isoform in the seed embryo [54]. Concurrently, PSAD1 contributes to the stability of this photosystem, and as a hydrophilic protein located in the stroma, it directly engages with ferredoxin within the electron transport chain [55, 56]. A recent report has identified FAHD1 as influencing seed longevity and dormancy [57]. Although FAHD-containing enzymes are not yet extensively studied in plants, they catalyze diverse reactions, including the hydrolysis of ß-diketones, decarboxylation, and isomerization [58–60]. Seeds of *Arabidopsis* that lacked FAHD1-a showed higher levels of antioxidants and a more reducing cellular environment, resulting in more resistance to seed aging [57].

During early imbibition, antioxidant protein activity decreases as a result of protein carbonylation [61], causing a transient rise in ROS, which act as signaling molecules to initiate germination [62]. Seed germination is precisely governed by the “oxidative window,” a state where ROS levels must be maintained within an optimal range to facilitate dormancy release and orchestrate germination signaling pathways [39, 63]. The accumulation of ROS also cleaves the wall polysaccharide to loosen the plant cell wall, enabling rapid water uptake [61].

### DNA damage and repair: gatekeepers of seed longevity

**Fig. 2:**
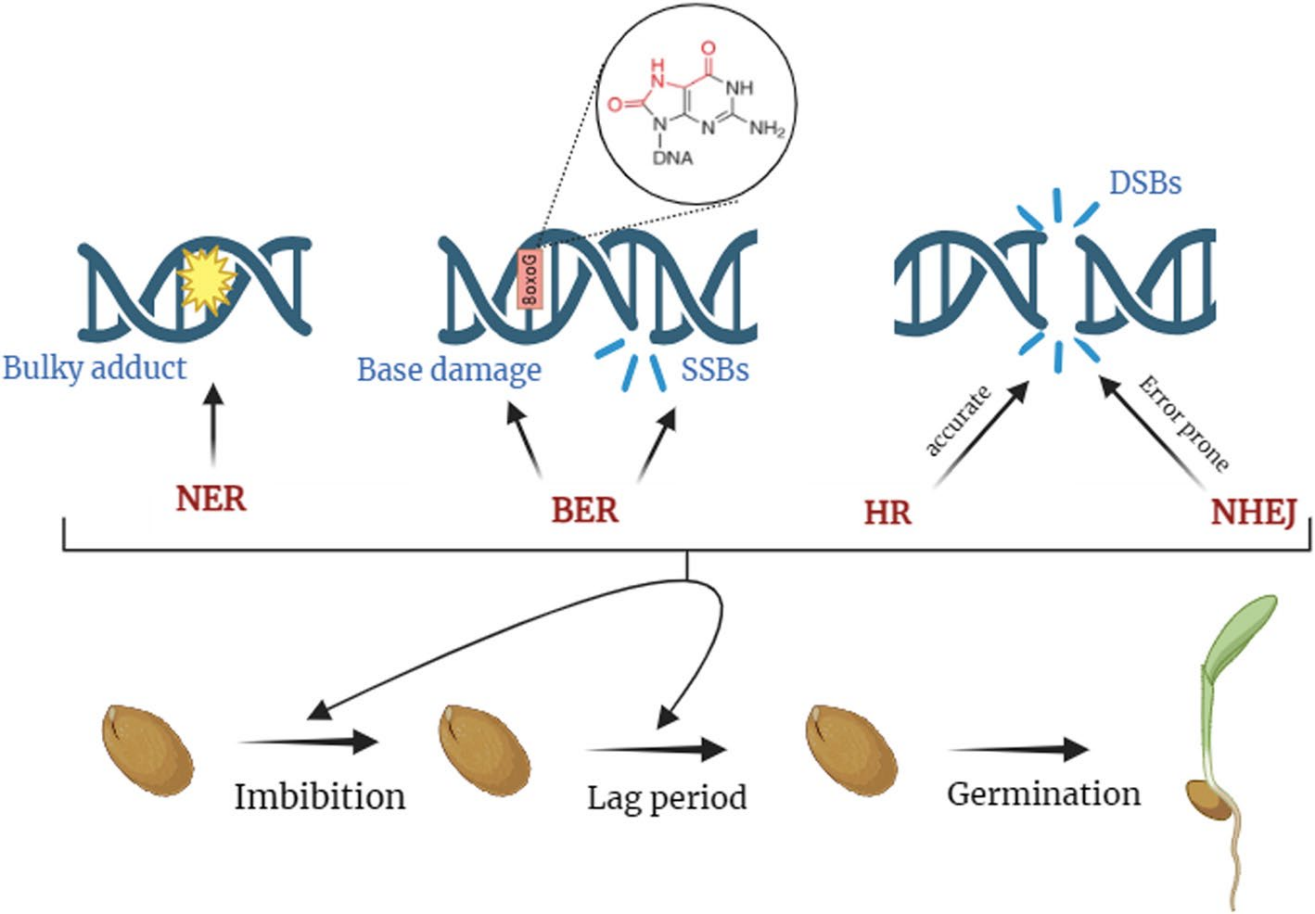
Schematic representation of the principal DDR pathways in seeds. NER addresses damage occurring on a single strand of the DNA duplex, particularly bulky adducts and lesions that impede RNA polymerase activity, resulting in faithful restoration of the duplex. BER focuses on removing damaged nucleotide bases, such as 8-oxoG, and repairing SSBs. DNA DSBs are repaired through mechanisms including HR and NHEJ pathways. HR repairs DSBs using a homologous sequence as a template, providing high-fidelity repair. NHEJ directly ligates the ends of DSBs without the need for sequence homology, constituting another critical pathway for DSB repair. Abbreviations: BER, base excision repair; DDR, DNA damage repair; DNA, deoxyribonucleic acid; DSB, double-strand breaks; HR, homologous recombination; NER, nucleotide excision repair; NHEJ, non-homologous end joining; oxoG, oxoguanine; RNA, ribonucleic acid; SSB, single-strand break

As previously elaborated, the process of desiccation serves as a mixed blessing for seeds. During this phase, the near-cessation of metabolic activity drastically slows the kinetics of DNA damage, thereby mitigating mutagenic processes. On the other hand, this quiescent state simultaneously disables the DNA repair machinery, rendering it vulnerable to the cumulative impact of unrepaired lesions. In over-extended storage, particularly under conditions of fluctuating or elevated humidity, subtle but persistent oxidative and hydrolytic assaults can escape repair, silently building a “molecular scar load” within the genome. The subsequent transition to hydration introduces further complexity, as rehydration reanimates metabolic processes while simultaneously eliciting a surge of ROS and mechanical stress at the chromatin level, thereby compounding pre-existing lesions [64]. During seed aging, base oxidation is the most common type of DNA lesion, with guanine being especially susceptible due to its low redox potential [65]. Guanine’s susceptibility to oxidation leads to the formation of mutagenic 8-oxoG, which can cause mispairing with adenine and compromise genetic integrity [66]. Base excision repair is the most prevalent pathway for addressing such damages (Fig. 2), which begins with DNA glycosylase enzymes that recognize and excise damaged bases, resulting in the formation of an abasic site [34, 67]. Subsequently, apurinic/apyrimidinic endonucleases or lyases process this site, allowing for DNA synthesis to fill the gap left by the removed base [68]. Notably, during the process of seed imbibition, an elevated expression of 8-oxoG DNA glycosylase 1 and formamidopyrimidine-DNA glycosylase enzymes in *Medicago truncatula* highlights the critical role of excision repair mechanisms in ensuring successful germination [69]. NER, which removes large DNA lesions and photoproducts, especially those generated by ultraviolet light, is another crucial repair pathway affecting seed viability (Fig. 2). Deficiency in the xeroderma pigmentosum group B protein, which facilitates DNA helicase activity within the NER pathway, resulted in compromised germination compared to wild-type seeds when subjected to hypochlorite treatment, a known inducer of oxidative DNA damage [70]. This finding suggests that the NER pathway is activated during seed imbibition and plays a critical role in maintaining seed viability.

DNA DSBs represent one of the most catastrophic forms of genome damage, essentially fragmenting the chromosome and threatening cell viability if left unrepaired [71–73]. In orthodox seeds, genomic stress is evidenced by extensive chromosome fragmentation, observable even in unaged, high-quality seeds, which results in elevated levels of chromosomal abnormalities, relative to other developmental stages [73]. In *Arabidopsis* seeds, the presence of chromosomal breaks is sufficient to slow or block germination, and failure to repair this damage prior to germination results in genome instability and low-vigor seedlings [74]. The capacity to detect and faithfully repair these lesions during the critical window of rehydration is therefore a determinant of viability and longevity, effectively deciding whether a genome can be “stitched back together” for successful germination. DSBs are repaired via either NHEJ or HR (Fig. 2). The importance of DNA repair in seed longevity has been substantiated through investigations into DNA ligase mutants, which are impaired in the NHEJ repair of DSBs. Specifically, mutations in DNA ligase 4 and DNA ligase 6, responsible for the canonical and alternative NHEJ repair pathways, respectively, render seeds exceptionally vulnerable to accelerated aging [75]. Intriguingly, a comprehensive genome-wide investigation into the genetic factors influencing seed longevity in *Arabidopsis* revealed a quantitative trait locus that aligned with the chromosomal position of LIG4 [76]. HR-directed repair of DSBs is also crucial in seeds, as demonstrated by gamma-irradiated maize rad51 mutants exhibiting delayed germination and increased seedling mortality compared to wild-type lines [77]. Waterworth et al. (2016) demonstrated that the DNA damage checkpoint kinase ATM regulates seed germination and maintains genome stability [40]. Seeds impaired in DNA damage repair exhibit heightened aging sensitivity, underscoring that efficient break repair is crucial for sustaining vigor, as enhanced repair capacity promotes seed longevity and aging resistance [78], thereby reinforcing the clear connection between DNA repair efficiency and seed longevity.

### RNA modification and loss of viability

Studies indicate that mature dry seeds of *Arabidopsis* and rice can germinate in the presence of transcription inhibitors. However, germination is inhibited when translation inhibitors are present, suggesting that the proteins necessary for germination are synthesized from pre-existing mRNA molecules within the mature dry seeds [79, 80]. The accumulation of mRNAs essential for rice seed germination during the maturation phase further reinforces this observation [81]. It has been proposed that the early translation of stored mRNAs enables seeds to rapidly resume their metabolic activity upon imbibition [82, 83]. However, cellular RNA is highly susceptible to oxidative damage [84]. Both ribosomal and messenger RNAs deteriorate during seed aging, making RNA integrity a sensitive indicator of seed viability loss during dry storage [85, 86]. Oxidative damage has been identified as a contributing factor to the fragmentation of mRNA observed in soybean seeds following extended storage periods exceeding two decades [87]. Oxidation of certain mRNAs, particularly those coding for genes with presumptive roles in cell signaling, was identified during the after-ripening process of sunflower seeds [88].

Recent studies have shown that RNA integrity number values decline linearly with time during seed storage, providing a robust quantitative marker for assessing seed age and viability in both crop and wild species [89, 90]. Impaired mRNA function hinders translation, and a reduction in translational activity in imbibed seeds has been associated with diminished seed longevity. This further underscores the critical importance of maintaining mRNA integrity and translational efficiency for successful seed germination. There is growing evidence that seeds use redox-regulated protective pathways and macromolecular repair mechanisms to slow down RNA degradation; antioxidants and RBPs help maintain stored mRNA functionality during aging and support rapid metabolic reactivation post-imbibition [33]. Many RBPs act as molecular chaperones, binding to specific motifs or structures within mRNA transcripts and physically shielding them from ROS generated during seed aging. For instance, glycine-rich RBPs are upregulated during seed development and have been implicated in improving mRNA stability during dehydration and aging [91]. Despite these advances, the precise molecular mechanisms underlying RNA protection and repair in seeds remain incompletely understood. Ongoing research is increasingly highlighting the essential roles of RBPs, redox-regulated pathways, and epigenetic modifications in maintaining RNA stability and functional integrity during seed storage and aging.

### Protein modifications and repair: the unsung heroes

Similar to DNA and RNA, proteins are susceptible to spontaneous and stress-induced damage, which frequently results in a decline in their functional capacity [92]. As previously detailed, the enzymatic activity of DNA repair proteins during imbibition is a critical determinant of seed longevity. Simultaneously, the components of the translational machinery must remain functional during dry storage to ensure efficient translation of stored mRNAs, which is essential for successful germination. Oxidation and carbonylation represent the principal modifications that impair protein function in aging seeds [61, 93]. These modifications can impair enzymatic activity, alter protein structure, and ultimately compromise cellular functions essential for germination and seedling establishment [11, 94], underscoring the critical role of protein repair enzymes in maintaining seed viability. The primary entities affected by these modifications are the abundant SSPs and metabolic enzymes [93]. Cruciferins are the most abundant SSPs in *Arabidopsis* that buffer the seed from oxidative stress, protecting important proteins required for seed germination and seedling formation. Cruciferin-deficient mutants are significantly more sensitive to oxidative stress [95].

Nevertheless, the proper functioning of SSPs, which serve as crucial reservoirs of amino acids for the developing embryo, is also contingent upon the prevention and repair of such modifications. The oxidative burden during aging facilitates the oxidation of methionine to MetSO, a prevalent form of protein damage that serves as a biomarker of aging across diverse organisms [96]. The accumulation of MetSO disrupts protein structure and catalytic activity, impeding the availability of amino acids and altering metabolic pathways during germination. MSR catalyzes the reduction of MetSO, thereby facilitating the repair of oxidized proteins through a reversible mechanism [97, 98]. MSR enzymatic activity is indispensable for the maintenance of seed protein function during storage and upon imbibition; in *M. truncatula* and alfalfa, higher natural MSR activity correlates with increased seed longevity, demonstrating conservation of this protective system in wild legumes [97, 99]. The *Arabidopsis* contains five MSRA genes and nine MSRB genes, whereas rice possesses three genes of each type. However, both MSRA and MSRB are recognized for their roles in enhancing plant tolerance to both biotic and abiotic stresses [100, 101]. Hazra et al. (2022) demonstrated that ascorbate peroxidase and PIMT are subject to MetSO modification in seeds, which impairs their functional capacity [102]. Rice MSRB5 forms a physical association with these proteins, reversing this modification to restore their functions and maintain seed vigor and longevity. Conversely, PIMT has been identified as crucial for salinity stress tolerance and overall seed longevity by repairing isoAsp accumulation in proteins [103]. IsoAsp residues, which result from the spontaneous deamidation of asparaginyl and glutaminyl residues, can disrupt protein structure and function, leading to the destabilization of storage proteins and enzymes and ultimately compromising seed viability. PIMTs directly repair these damaged sites, allowing their conversion to normal residues and thereby restoring proper protein function. The repair of these isoAsp modifications by PIMTs maintains protein integrity, which is essential for preserving enzyme activity and structural components critical for successful germination and seedling development [104]. For instance, PIMT mediates the repair of isoAsp modifications in the enolase 2 protein, thereby safeguarding its essential cellular functions throughout seed maturation and storage in rice [105]. Further reports suggest that overexpression of the chickpea PIMT genes, *CaPIMT1* and *CaPIMT2*, led to greater longevity in *Arabidopsis* seeds [106]. Therefore, the synergistic action of MSR and PIMT repair systems is fundamental to seed longevity by reversing specific oxidative and age-related protein damage, thereby preserving the functional integrity of a wide array of proteins essential for germination and stress resilience.

### Protective proteins: the passive defenders

The synthesis and accumulation of LEA proteins and HSPs constitute a critical hallmark of seed maturation (Fig. 1). LEA proteins are distinguished by a high proportion of glycine, a limited presence of cysteine and tryptophan, and a prevalence of alanine, glutamate, lysine/arginine, and threonine [107]. These primary structure characteristics contribute to the stability of LEA proteins across a wide temperature spectrum and high hydration levels. During cellular dehydration, LEA proteins function as molecular chaperones, contributing to the structural integrity of other proteins and cell membranes through extensive hydrogen bond formation, thereby stabilizing denatured proteins and facilitating their refolding [108]. The plant kingdom features a considerable number of LEA genes, *Arabidopsis* alone containing up to 51, which are categorized into seven groups based on their Pfam domains [109]. Based on the Pfam protein domain database, LEA proteins are classified into eight distinct families: Dehydrins (PF00257), LEA-1 (PF03760), LEA-2 (PF03168), LEA-3 (PF03242), LEA-4 (PF02987), LEA-5 (PF00477), LEA-6 (PF10714), and SMPs (PF04927) [108, 110]. The precise molecular roles of LEA proteins in contributing to seed longevity and the underlying reasons for their varied expression in desiccated seeds are not yet fully understood. However, reports suggest a significant downregulation of three seed-specific dehydrins in *Arabidopsis* correlates with reduced seed viability during storage [110]. Homologs of LEA proteins are found in both the cytosol and nucleus, while PM10 is located in the vacuole and endoplasmic reticulum, and LEAm in the mitochondria [111]. LEA proteins are anticipated to perform a variety of functions during the maturation of seeds. Given their hydrophilic characteristics and capacity to buffer hydration levels, specific LEA proteins are speculated to play a role in regulating water loss during seed maturation [112]. Mutations in the *Arabidopsis* EM6 gene have been associated with impaired maturation drying [113]. During progressive desiccation, LEA proteins are postulated to shield cells from the concomitant reduction in water potential by stabilizing proteins and inhibiting their aggregation [114]. The functional interaction between dehydrins and phospholipids indicates LEA proteins may influence membrane properties [115]. LEA proteins may also act to buffer any increase in ion concentration, particularly Fe2 +, which can catalyze the production of ROS [116]. Additionally, during advanced stages of drying, these proteins may contribute to vitrification in association with sucrose and oligosaccharides [117, 118].

In *M. truncatula*, transcriptomic analysis revealed 12 seed-specific LEA transcripts, while proteomic investigations further detected LEA polypeptides corresponding to six additional genes [119]. Notably, this research also reported a 30-fold enhancement in longevity correlating with the accumulation of four LEA polypeptides, which comprised 35% of the total LEA proteins in mature seeds. Subsequently, a temporal study showed that only a few LEA proteins accumulated at the onset of desiccation tolerance, while most increased markedly during late maturation. Interestingly, their proteins appeared much later than their transcripts, which were detected 10 to 20 days earlier [120]. This indicates that LEA protein abundance coincides with the final phase of water loss, and the pronounced temporal lag between transcript accumulation and protein presence points toward post-transcriptional control of LEA protein expression. This prompts a crucial inquiry: What molecular mechanisms underlie the delayed translation and/or stabilization of LEA proteins during the late maturation phase, and what role do these processes play in conferring desiccation tolerance and seed longevity? It is plausible that this regulation facilitates the coordinated production of LEA proteins at the appropriate stage, thereby enhancing seed storability.

Similar to LEA proteins, HSPs also accumulate during the late stages of seed maturation and are found in their native form within dry seeds. Eukaryotic HSPs are classified into five distinct groups based on their molecular weights: Hsp100, Hsp90, Hsp70, Hsp60, Hsp40, and the sHSPs. The sHSPs constitute the largest and most heterogeneous group within the entire HSP superfamily, with molecular weights ranging from 12 to 43 kDa, and they are distinguished by a highly conserved C-terminal crystalline domain of 80 to 100 amino acids [121]. sHSPs function as ATP-independent chaperones, preventing protein aggregation and maintaining proteins in a folding-competent state, with the capacity to refold misfolded proteins either independently or in conjunction with other ATP-dependent chaperones [122, 123]. The accumulation of sHSP transcripts during late maturation, despite the absence of heat stress, highlights their potential role in safeguarding proteins from denaturation and aggregation under the extreme desiccation and metabolic quiescence that seeds undergo [124]. Kaur et al. (2015) reported that the expression of rice HSP18.2 enhanced seed vigor and longevity by mitigating the accumulation of detrimental ROS in stored seeds [122]. The expression of HSP genes is controlled by HSFs, a large and diverse family of transcription factors that serve as the terminal regulators of the stress-responsive signaling pathway leading to HSP induction. During seed maturation, HSP expression is not induced by stress; rather, it is developmentally regulated by the seed-specific HSFA9, which upregulates HSPs to promote desiccation tolerance and ensure long-term seed viability. Furthermore, the transcriptional activation of HSFA9 is governed by ABI3, as evidenced by the absence of HSFA9 transcripts and proteins in ABI3 knockout lines [124].

### Functional synergy of redox, repair, and protective pathways

**Fig. 3:**
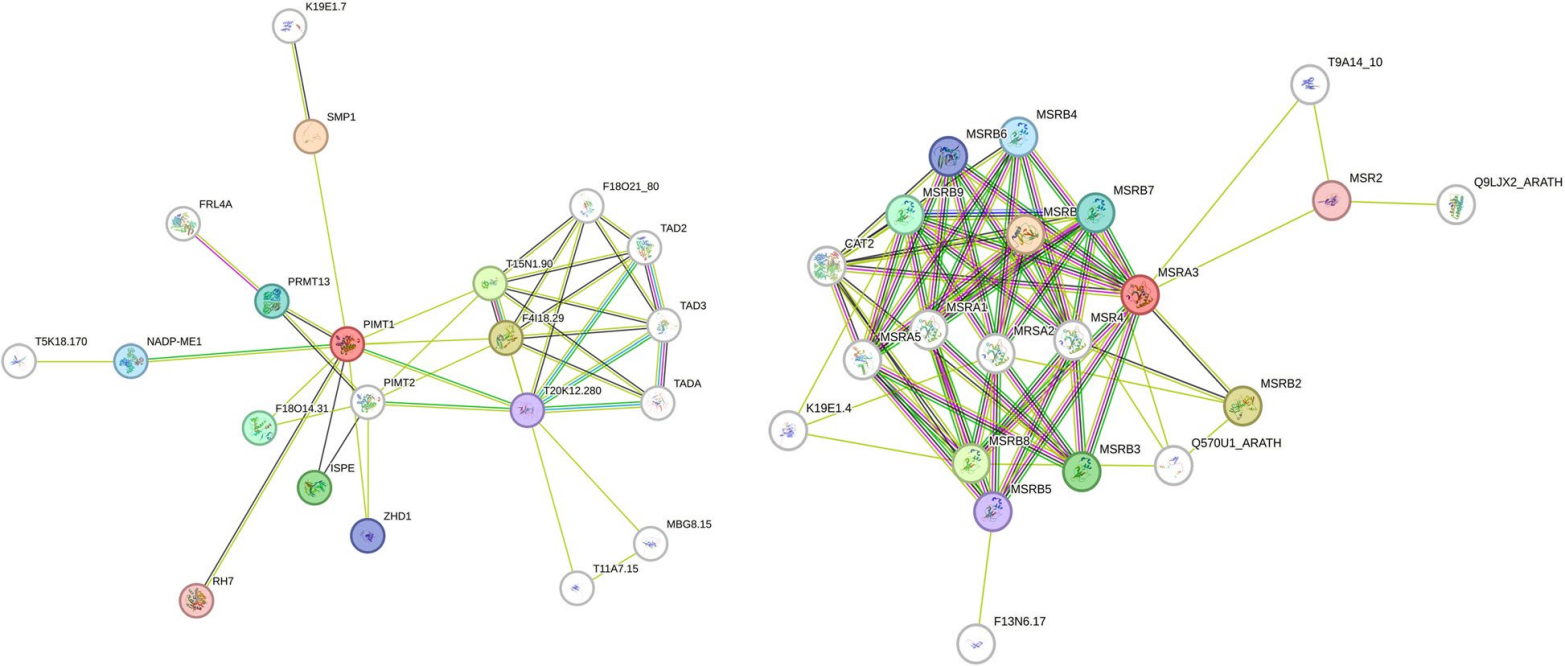
Protein interaction network of PIMT and MSR. PPIs were identified using STRING network analysis (version 12.0). The depicted interaction networks show that both repair proteins in *Arabidopsis* engage with a range of partners. Nodes represent proteins and edges indicate predicted interactions. Representative enriched interactors for PIMT1 and PIMT2 include *SMP1*, *F4I18.29*, *T15N1.90*, *ISPE*, *F18O14.31*, *PRMT13*, *NADP-ME1*, *ZHD1*, *T20K12.280*, and *RH7*. For the MSR family, notable interactions were observed with *CAT2*, *F13N6.17*, *Q9LJX2_ARATH*, *T9A14_10*, and uncharacterized proteins (*K19E1.4*, *Q570U1_ARATH*). A comprehensive list of identified PIMT interacting partners, along with their confidence scores, is provided in Table 1, and MSR interacting partners are listed in Table 2. Abbreviations: CAT2, catalase-2; F13N6.17, antigenic heat-stable protein; F18O14.31, glutathione S-transferase family protein; F4I18.29, tRNA (adenine(58)-N(1))-methyltransferase non-catalytic subunit TRM6; ISPE, 4-diphosphocytidyl-2-C-methyl-D-erythritol kinase; MSR, methionine sulfoxide reductase; NADP-ME1, nicotinamide adenine dinucleotide phosphate–dependent malic enzyme 1; PIMT, protein L-isoaspartyl methyltransferase; PPI, protein–protein interaction; PRMT13, histone-arginine methyltransferase 13; Q9LJX2_ARATH, plant invertase/pectin methylesterase inhibitor superfamily protein; RH7, DEAD-box ATP-dependent RNA helicase 7; RNA, ribonucleic acid; SMP1, seed maturation protein 1; T15N1.90, tRNA (adenine(58)-N(1))-methyltransferase; T20K12.280, phosphatidylinositol N-acetyglucosaminlytransferase subunit P-like protein; T9A14_10, magnesium transporter NIPA7; tRNA, transfer RNA; ZHD, zinc finger–homeodomain protein

**Fig. 4:**
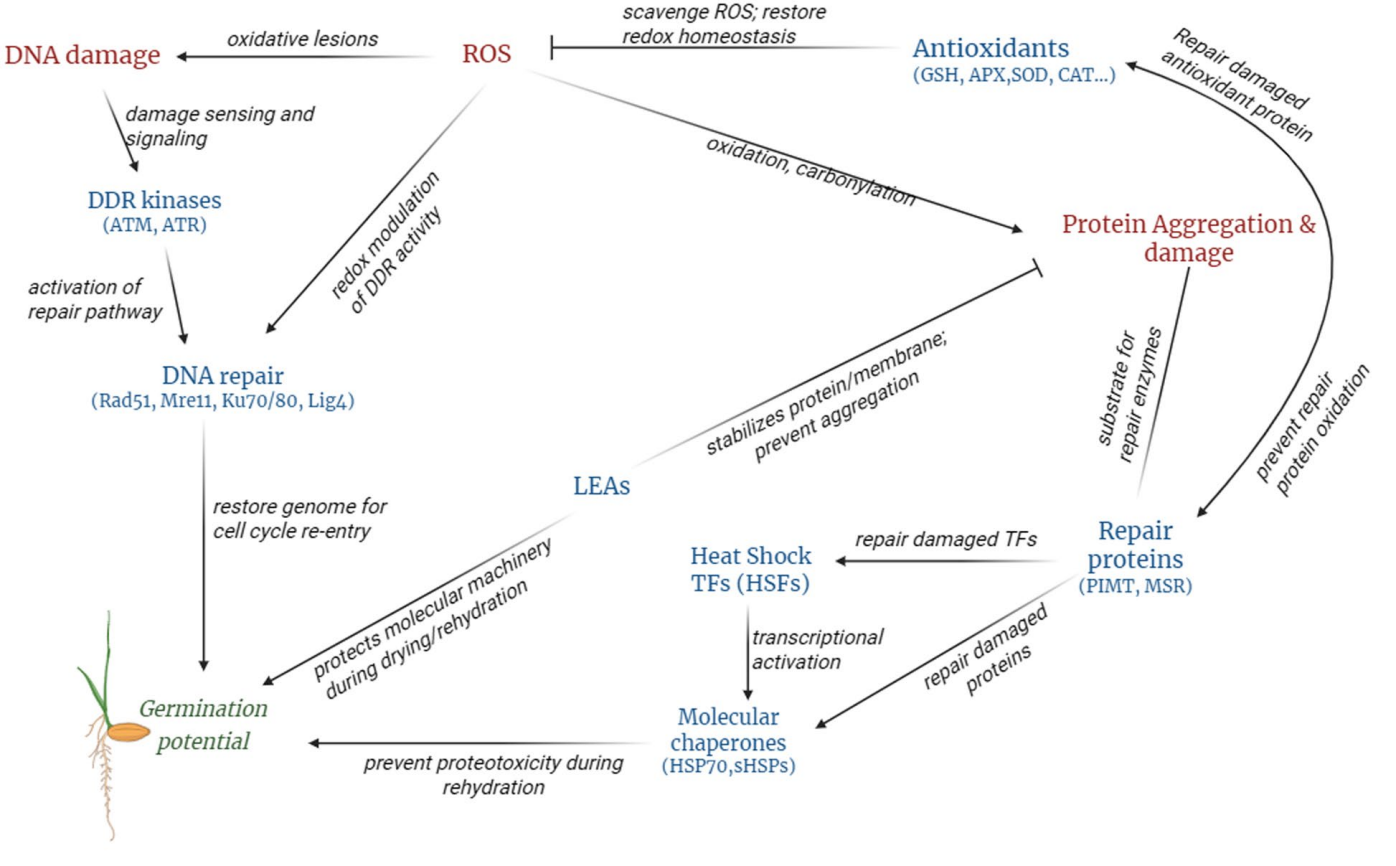
The intricate interplay between redox homeostasis, DNA and protein repair mechanisms, HSPs, and LEA proteins in regulating seed germination and longevity. The diagram elucidates the complex mechanisms underlying seed viability throughout desiccation, storage, and imbibition. ROS act as damaging agents, leading to oxidative lesions in DNA and proteins. Cellular antioxidant systems (GSH, APS, SOD, CAT) mitigate ROS to maintain redox homeostasis. Upon rehydration, DNA damage accumulated during seed maturation and storage activates DDR signaling through DDR kinases (ATM/ATR), which in turn promotes double-strand break repair via homologous recombination (RAD51, MRE11, NBS1) and non-homologous end joining (KU70/80, LIG4). Protein oxidation and aggregation are counteracted by protein repair enzymes such as PIMT and MSR, which act synergistically with molecular chaperones (HSP70, small HSPs) to refold or stabilize damaged proteins. Heat-shock transcription factors transcriptionally activate HSPs and cooperate with proteins to preserve protein and membrane integrity during desiccation and rehydration. The protein repair system also restores the function of damaged heat-shock transcription factors, thereby preserving their transcriptional activity. Collectively, these integrated systems establish a comprehensive network that restores genomic integrity, prevents proteotoxicity, and sustains cellular homeostasis, ultimately facilitating successful seed germination and extending seed longevity. Abbreviations: ATM, ataxia-telangiectasia mutated; ATR, ataxia telangiectasia; CAT, catalase; DDR, DNA damage repair; DNA, deoxyribonucleic acid; GSH, glutathione; HSP, heat-shock protein; LEA, late embryogenesis abundant; LIG4, ligase 4; MRE11, meiotic recombination 11; MSR, methionine sulfoxide reductase; PIMT, protein L-isoaspartyl methyltransferase; ROS, reactive oxygen species; SOD, superoxide dismutase

Seed viability contingent upon the re-establishment of active metabolism involves the recruitment of functional proteins through three avenues: (i) using proteins from the stored proteome that have survived maturation desiccation and prolonged dry storage, (ii) translating stored mRNA produced during seed maturation, or (iii) synthesizing new mRNA via de novo transcription. Those proteins essential for the translational apparatus are the “Achilles’ heel” of seed longevity and must be present in the stored proteome that has survived maturation, desiccation, and subsequent rehydration. Proteins involved in the translational process were identified as key substrates for PIMT in seeds [125] (Fig. 3). Correspondingly, isoAsp formation leads to a loss of activity of ATP-dependent, DEAD-box RNA helicase, an effect that can be reversed by PIMT in *Arabidopsis* [126]. Another target of PIMT, not directly involved in translation, is SMP1, which is instrumental in seed maturation and retention dormancy during high-temperature fluctuations (Fig. 3; Table 1). The restoration of an SMP1 protein by PIMT1 suggests that LEA proteins may require PIMT1-mediated protection against isoAsp formation to preserve their function, potentially forming a component of an integrated network of protein-protective mechanisms present in seeds. In *Arabidopsis*, PIMT also repairs superoxide dismutase and catalase, antioxidant proteins susceptible to isoAsp accumulation under thermal stress [103] (Fig. 4). Similarly, the PIMT isoforms in the wild rice variety *Oryza coarctata* have been observed to interact with and repair antioxidant enzymes that have sustained isoAsp damage, thereby preserving their functional characteristics [127]. Interestingly, catalases are notably vulnerable to oxidative damage due to their high content of methionine residues. MSRs may reverse the oxidation of methionine, thereby restoring catalase activity [128] (Fig. 3; Table 2). Notably, recent comparative genomic studies reveal a greater diversity of MSR isoforms in wild plants, suggesting evolutionary selection for protein repair systems that maintain seed viability in challenging environments [129, 130]Previous studies have firmly established that the acquisition of seed desiccation tolerance is mediated through ABA signaling, which is facilitated by elevated ABA levels and the coordinated expression and activity of key ABA signaling regulators during seed maturation [131, 132]. ABI3 controls the expression and accumulation of the antioxidant and LEA proteins during the late maturation phase, equipping seeds for prolonged quiescence and oxidative stress resistance [133]. Recent mechanistic insights have further revealed the pivotal role of protein repair systems in safeguarding the integrity of these signaling networks. The repair protein PIMT counteracts isoAsp accumulation in ABI transcription factors (ABI3, ABI4, and ABI5) and modulates their target gene expression during seed maturation, thereby preserving their transactivation activity essential for seed desiccation tolerance and longevity [134]. The interaction network of PIMT indicated its association with ZF-HD, a plant-specific transcription factor critical for development and stress tolerance (Fig. 3; Table 1). PIMT may sustain the function of ZF-HD transcription factors, thereby ensuring the continued regulation of stress-protective genes (e.g., HSPs, LEA proteins, and antioxidants) during the later stages of maturation and upon imbibition. Furthermore, recent evidence demonstrates that PIMT-mediated repair of isoAsp modification in the heat-shock transcription factor *Os*HSFC1b positively regulates seed vigor and key agronomical seed traits [135]. These interplays suggest a broader function for protein repair machinery in seeds, not only preserving the structural fidelity of the proteome but also underpinning the preservation of essential gene regulatory networks that drive successful germination and stress resilience (Fig. 4).

**Table 1:** Lists the interacting partners of *Arabidopsis* PIMTs, identified using STRING database version 12.0, with PIMT1 as the source protein. The confidence score reflects the predicted likelihood of interaction, with higher values indicating greater support from experimental data, curated databases, or computational predictions

Interactor	Description	Confidence Score
PIMT2	Protein-L-isoaspartate O-methyltransferase 2	0.738
ZHD1	Zinc finger homeodomain–like protein 1	0.702
DP-ME1	NADP-dependent malic enzyme 1	0.696
MBG8.15	DTW domain-containing protein	0.680
F4I18.29	tRNA (adenine(58)-N(1))-methyltransferase non-catalytic subunit TRM6	0.684
TADA	tRNA(adenine(34)) deaminase, chloroplastic	0.660
F18O21_80	tRNA (guanine(37)-N1)-methyltransferase 1	0.659
T15N1.90	tRNA (adenine(58)-N(1))-methyltransferase	0.652
PRMT13	Probable histone-arginine methyltransferase 1.3	0.669
T11A7.15	DTW domain-containing protein	0.665
ISPE	4-diphosphocytidyl-2-C-methyl-D-erythritol kinase, chloroplastic	0.642
TAD3	tRNA-specific adenosine deaminase TAD3	0.638
K19E1.7	Late embryogenesis abundant protein 50	0.635
T20K12.280	Phosphatidylinositol N-acetyglucosaminyltransferase subunit P-like	0.622
RH7	DEAD-box ATP-dependent RNA helicase 7	0.610
T5K18.170	Uncharacterized protein	0.609
SMP1	Seed maturation protein 1	0.607
FRL4A	FRIGIDA-like protein 4a	0.605
TAD2	tRNA-specific adenosine deaminase TAD2	0.602
F18O14.31	Glutathione S-transferase family protein	0.598

Abbreviations: NADP, nicotinamide adenine dinucleotide phosphate; PIMT, protein L-isoaspartate methyltransferase; tRNA, transfer ribonucleic acid

**Table 2:** Lists the interacting proteins of the *Arabidopsis* MSR family, identified using the STRING database version 12.0, with MSRA3 as the source protein. The confidence score reflects the predicted likelihood of interaction, with higher values indicating greater support from experimental data, curated databases, or computational predictions

Interactor	Description	Confidence Score
MSR2	Mannan synthesis-related 2, glycosyltransferase	0.776
Q570U1_ARATH	Uncharacterized protein	0.684
MRSA2	Peptide methionine sulfoxide reductase A2	0.672
K19E1.4	Uncharacterized protein	0.487
Q9LJX2_ARATH	Plant invertase/pectin methylesterase inhibitor superfamily	0.470
T9A14_10	Magnesium transporter NIPA7	0.468
F13N6.17	Antigenic heat-stable protein	0.465
CAT2	Catalase-2	0.429

Abbreviation: MSR, methionine sulfoxide reductase

In this context, the capacity for efficient DNA repair represents another critical determinant of seed longevity and vigor. DNA damage caused by desiccation, ROS accumulation, and storage-associated aging can hinder de novo transcription upon rehydration, ultimately impairing the expression of proteins essential for seedling establishment (Fig. 4). Recent evidence demonstrates that protein repair systems such as PIMT also extend the functional lifespan of DNA repair proteins, ensuring that DNA lesions accumulated during storage and aging can be rapidly and efficiently resolved upon imbibition [136]. In DT seeds, changes in chromatin dynamics are crucial for protecting genomic integrity. The interaction of PIMT with protein arginine N-methyltransferases—a chromatin modulator that methylates histone-arginine residues [137] (Fig. 3; Table 1)—suggests a mechanistic link that couples protein repair with DNA repair to promote seed viability. Additionally, PRMT family members such as PRMT5 coordinate the alternative splicing and expression of key DNA repair genes, including TIP60, RAD51, and TP53, which are crucial for genome integrity and ensuring successful germination [138]. Consequently, protein repair systems help preserve the activity of DNA repair proteins and prolong their lifespan, allowing seeds to quickly and effectively repair DNA damage that has accumulated during aging, upon imbibition. On the other hand, LEA proteins, which are intrinsically disordered, refold into structures with a higher proportion of amphipathic alpha-helices upon dehydration [139–141]. These adaptable structural features enhance their binding affinity to DNA and RNA, suggesting that LEA proteins contribute to the stabilization of genetic material and ribonucleoprotein complexes during the desiccation process. The cumulative insights from these studies underscore that the integration of protein repair, chromatin modulation, redox, and protective proteins create a synergistic network that safeguards genomic integrity and ensures successful seedling establishment by maintaining the capacity for DNA repair and essential transcription upon rehydration.

## Conclusions and outlook

Seed longevity, a critical agronomic trait influencing germination success and crop productivity, plays a pivotal role in global food security. This characteristic is maintained through a complex interplay of redox homeostasis, DNA repair systems, and protective proteins, which collectively mitigate the adverse effects of desiccation and dormancy. This intricate biological interplay underscores the sophisticated mechanisms that have evolved to ensure successful seed germination across diverse environmental conditions. Understanding these mechanisms enables the development of molecular biomarkers for seed storage stability, guides the selection of genotypes with superior repair capacity for breeding, and helps refine storage or priming protocols to sustain viability under various conditions. A more comprehensive understanding of the molecular interplay among these pathways is essential for advancing seed longevity. Identifying key regulatory nodes that integrate redox homeostasis and repair pathways will provide deeper mechanistic insight and reveal targets for biotechnological interventions. Such advances will drive the development of innovative strategies to enhance seed vigor, extend shelf life, and bolster agricultural resilience in the face of climate challenges, supporting sustainable food production worldwide.

## Data Availability

Not applicable.

## Abbreviations

8-oxoG: 8-Oxoguanine
ABA: Abscisic acid
ABI: ABA-insensitive
ATM: Ataxia-telangiectasia mutated
ATP: Adenosine triphosphate
DHAR: Dehydroascorbate reductase
DNA: Deoxyribonucleic acid
DSB: Double-strand breaks
DT: Desiccation-tolerant
FAHD1: Fumarylacetoacetate hydrolase domain-containing protein 1
GSH: Reduced glutathione
GSSG: Oxidized glutathione
HR: Homologous recombination
HSF: Heat-shock factor
HSFA9: Heat-shock transcription factor 9
HSP: Heat-shock protein
isoAsp: Isoaspartyl
LEA: Late embryogenesis abundant
MetSO: Methionine sulfoxide
mRNA: Messenger ribonucleic acid
MSR: Methionine sulfoxide reductase
NER: Nucleotide excision repair
NHEJ: Non-homologous end joining
PIMT: Protein L-isoaspartyl methyltransferase
PSAD: Photosystem I subunit
RBP: RNA-binding protein
RFO: Non-reducing oligosaccharide
ROS: Reactive oxygen species
sHSP: Small heat-shock protein
SMP: Seed maturation protein
SSP: Seed storage protein
ZF-HD: Zinc finger–homeodomain
